# Transferencia embrionaria única: estrategia clave para reducir el riesgo de embarazo múltiple en reproducción humana asistida

**DOI:** 10.1515/almed-2020-0095

**Published:** 2021-02-08

**Authors:** Pilar Reimundo, Javier M. Gutiérrez Romero, Tamara Rodríguez Pérez, Ernesto Veiga

**Affiliations:** Laboratorio de Reproducción Asistida y Andrología, Área de Bioquímica Clínica, Laboratorios Clínicos Vall d’Hebron, Hospital Universitario Vall d’Hebron, Barcelona, España; UGC Laboratorios Clínicos, Hospital Universitario Puerta del Mar, Cádiz, España; Laboratorio de Andrología y Técnicas de Reproducción Asistida, Servicio de Análisis Clínicos, Hospital Universitario La Paz, Madrid, España; Laboratorio Central, Complejo Hospitalario Universitario de Santiago de Compostela (CHUS), SERGAS, Hospital Clínico Universitario de Santiago de Compostela, Santiago de Compostela, España

**Keywords:** embarazo múltiple, fecundación *in vitro*, infertilidad, parto múltiple, técnicas de reproducción asistida, transferencia embrionaria única electiva

## Abstract

El objetivo principal en los inicios de la reproducción humana asistida (RHA) era conseguir la gestación. Las tasas de éxito eran bajas y, por ello, las transferencias embrionarias múltiples se convirtieron en una práctica normal alcanzando tasas de embarazo múltiple hasta veinte veces superiores a las naturales. El embarazo múltiple está asociado a un mayor riesgo de complicaciones para la salud que un embarazo único, tanto para la madre como para los bebés. A los costes en salud deben sumarse también los costes económicos y los riesgos psicosociales, implicando por tanto un elevado coste socio-sanitario. En la actualidad, las tasas de éxito en RHA han mejorado enormemente gracias, en parte, a importantes avances del laboratorio como el cultivo embrionario hasta blastocisto y la vitrificación. Asimismo, existen diversas herramientas de asesoramiento, políticas sanitarias y económicas que han demostrado, tras su aplicación en varios países, su efectividad en el aumento de la práctica de la transferencia embrionaria única y en el descenso de las tasas de embarazo múltiple, garantizando unas tasas de éxito satisfactorias. Por todo ello, la transferencia embrionaria única se plantea como la estrategia de elección en RHA para conseguir un recién nacido vivo sano a término en casa.

## Introducción

Desde que en 1978 naciese el primer bebé mediante Técnicas de Reproducción Asistida (TRA), su uso y desarrollo se ha elevado enormemente, suponiendo un antes y un después para las parejas que presentan dificultades para concebir naturalmente [1]. Actualmente, el 2,6% de los recién nacidos en Europa [2] y más del 9% en España [3], son fruto de las TRA.

En sus comienzos, teniendo como objetivo central lograr el deseado embarazo, se intentaban maximizar las posibilidades de éxito transfiriendo varios preembriones, siendo habitual la transferencia de dos, tres o incluso cuatro por intento [4]. Esta ha sido una práctica rutinaria en numerosos países ya que la legislación durante las dos primeras décadas de desarrollo de las TRA así lo permitía. Concretamente en España, la Ley 35/1988 sobre TRA no ponía límites respecto al número de preembriones a transferir, quedando sujeto a criterio clínico. Ya en 2003 se publicó la Ley 45/2003, que modificaba la anterior, y autorizaba la transferencia de un máximo de tres preembriones por intento. Desde entonces, numerosos estudios han puesto el foco en el crecimiento exponencial de la tasa de embarazo múltiple en TRA y especialmente del embarazo gemelar, que en aquél momento se situaba en España en torno al 26% [5]. El embarazo múltiple representa la complicación iatrogénica más frecuente tras la aplicación de las TRA y supone un factor de riesgo en comparación con el embarazo simple, por asociarse a una mayor tasa de mortalidad y morbilidad materna y a problemas perinatales como el parto pretérmino y el bajo peso al nacer [6].

## Materiales y métodos

La bibliografía empleada para elaborar la presente revisión se obtuvo en el repositorio en línea Entrez Pubmed (US National Library of Medicine, National Institute of Health; http://www.ncbi.nlm.nih.gov/pubmed/). Las referencias se identificaron mediante una búsqueda que incluyó los siguientes términos: infertilidad, fecundación *in vitro* (FIV), transferencia embrionaria múltiple, transferencia de embrión único (SET), transferencia electiva de embrión único (eSET), gestación múltiple, gestación gemelar, segmentación embrionaria, blastocisto, vitrificación, tecnología time-lapse, test genético preimplantacional y TRA. Tras consultar las referencias bibliográficas de los artículos seleccionados mediante búsqueda primaria, se identificaron estudios relevantes adicionales. Como resultado, se analizaron un total de 84 artículos científicos publicados y revisados por pares que cumplieron los criterios indicados.

## Marco legal de la política de transferencia embrionaria en la Unión Europea (UE)

Así como existe una normativa común en la UE con respecto al uso (adquisición, almacenamiento, transporte, trazabilidad) de tejidos y células reproductoras y a su cribado infeccioso, no existe una legislación común aplicable a los procedimientos que se realizan en las clínicas de RHA [7], [8]. Los estados miembros son así libres de dictar su propia legislación, existiendo en todos ellos diferentes regulaciones que se actualizan en función de los avances técnicos y de la cobertura pública o no de los tratamientos. Por otra parte, en países como la India, Japón o EE. UU. no existe una legislación nacional en este sentido [9]. En casi todos los países, sin embargo, existen guías de buena práctica clínica elaboradoras por las Sociedades Científicas a nivel Nacional o Internacional que complementan las distintas legislaciones.

Con respecto a la transferencia embrionaria, la mayoría de países de la UE establece un límite legal sobre el número de embriones a transferir por intento, pero los escenarios varían enormemente. En algunos países solo se permite uno (Austria o Bélgica en menores de 36 años) y en otros hasta tres, según la voluntad de los pacientes y el consejo del equipo clínico (España o Alemania, aunque en Alemania se recomienda un máximo de dos en menores de 37 años). En la mayoría de países, aunque se recomiende la SET, hay límites edad dependiente. En otros, como Francia y Suecia, no se permite transferir nunca más de dos embriones. Y en algunos, como Bélgica, la financiación pública está supeditada a la política de transferencia embrionaria [10]. En Bulgaria, la ley aplica criterios concretos como edad de la mujer, número de intentos fallidos y estadio embrionario. En el extremo contrario, en República Checa, no existe ninguna ley sobre el número máximo de embriones a transferir, aunque la mayoría de clínicas recomiendan transferir uno o dos.

## Recomendaciones de las sociedades científicas sobre el número de embriones a transferir

**Figura 1:**
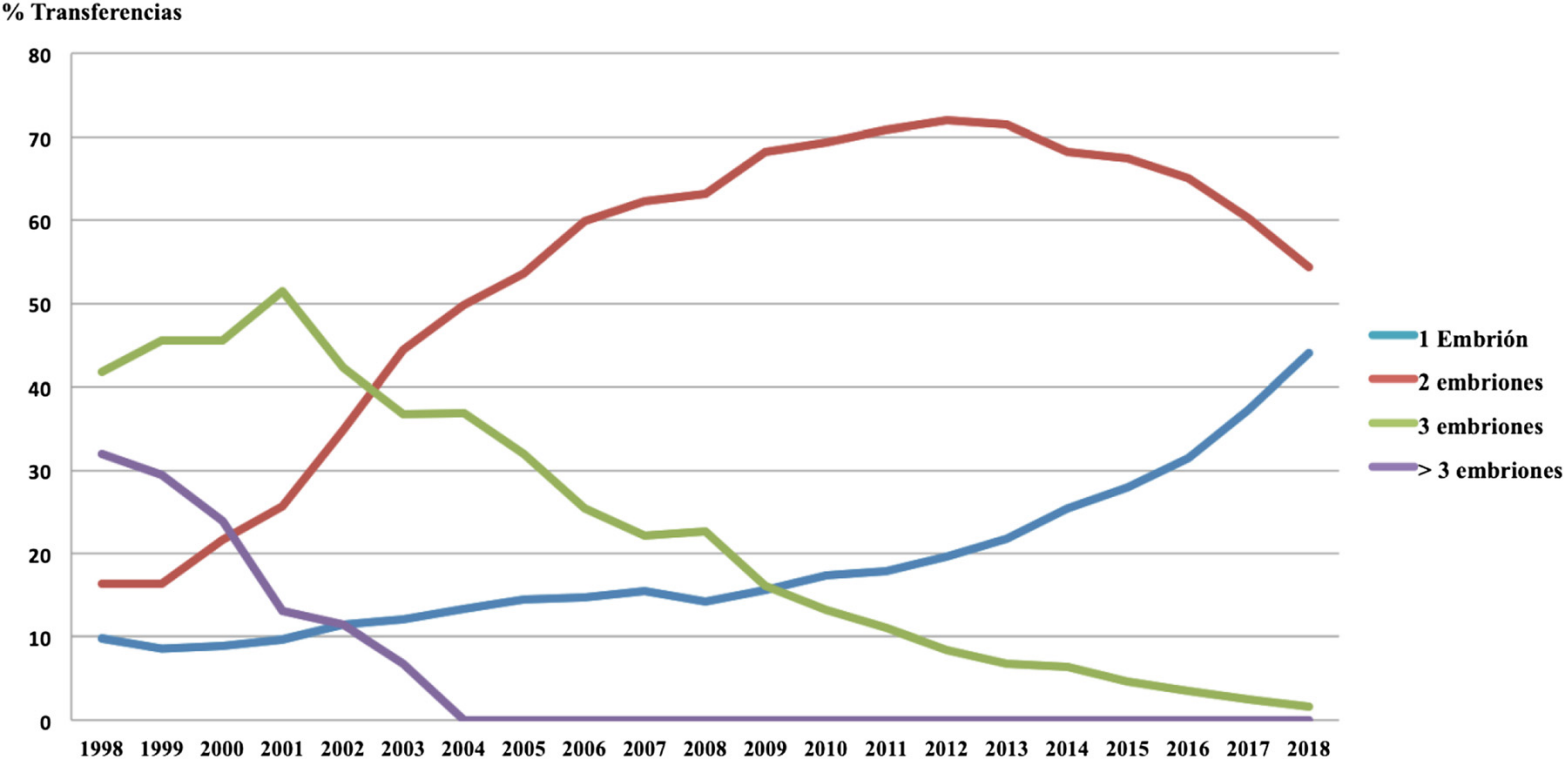
Evolución de la política de transferencia embrionaria en fresco en España entre los años 1998 y 2018, según datos del Registro Nacional de Actividad 2018-Registro SEF.

Con el ánimo de reducir el elevado porcentaje de gestaciones múltiples en TRA, las principales sociedades científicas han tratado de informar y concienciar sobre los efectos de la transferencia embrionaria múltiple. En 2002, la Sociedad Europea de Reproducción Humana y Embriología (ESHRE) revisó los riesgos de las TRA concluyendo que el objetivo de los tratamientos de FIV es el nacimiento de un único bebé sano y que, por tanto, la gestación múltiple se considera una complicación de los mismos [11]. Sociedades americanas como la Sociedad para la Tecnología de Reproducción Asistida (SART) y la Sociedad Americana de Medicina Reproductiva (ASRM) comenzaron también entonces a estudiar la importancia de minimizar el número de embriones transferidos, representando la SET en 2002 solamente el 1% del total de transferencias realizadas en EEUU [12], [13]. En esas mismas fechas en España, las transferencias en fresco de tres embriones representaron el 42,3%, las de dos embriones el 34,9% y las de un embrión el 11,4% mientras que en 2018 se situaron en un 1,6%, 54,4% y 44%, respectivamente (Figura 1) [3], [5].

En referencia a los pacientes, según varios estudios, muestran gran aceptación del embarazo gemelar y esta crece según aumenta el tiempo de infertilidad o los fracasos en tratamientos anteriores. Otros trabajos, sin embargo, demuestran que un mayor conocimiento de los posibles riesgos de un embarazo múltiple lo convierten en una opción menos deseable y hace que la SET sea mejor aceptada [14]. Actualmente, la creciente concienciación de los profesionales así como la mejora en la tecnología de laboratorio han hecho que la mayoría de centros opten por ofrecer la eSET [15].

## Mejoras y avances en el laboratorio que permiten eSET

### Cultivo hasta blastocisto

Mediante eSET se transfiere un embrión seleccionado por su mayor calidad de entre todos los embriones obtenidos. La transferencia puede hacerse en estadio de células (días 2–3 de desarrollo), mórula (día 4) o blastocisto (días 5-6-7), aunque cada vez más laboratorios optan por el estadio de blastocisto ya que permite una mejor selección [16]. El blastocisto es la estructura embrionaria con mayor celularidad y complejidad obtenida tras cultivo en el laboratorio, posterior a la fecundación y previo a la implantación en el útero. Se trata de un estadio avanzado que ha demostrado competencia *in vitro* para desarrollarse y diferenciarse en una *masa celular interna* que potencialmente dará lugar al futuro bebé y un *trofoectodermo* que formará la placenta. Este nivel de desarrollo, junto a una valoración cualitativa según características morfológicas, permiten seleccionar el embrión con mayor potencial implantatorio [17]. Así pues, el cultivo hasta blastocisto, comúnmente llamado cultivo largo, es una práctica cada vez más habitual. Su implementación requiere gran control y monitorización de las condiciones y procedimientos de laboratorio, lo cual ha sido posible gracias a la mejora en los medios de cultivo y los controles de calidad así como al desarrollo de incubadores trigas [18].

Los incubadores convencionales utilizados en la década de los 80 tenían temperatura y CO_2_ regulados pero estaban compartimentados solo parcialmente, de forma que su apertura modificaba las condiciones en todo el incubador. La temperatura se establece a 37 °C para simular las condiciones del organismo y el control del CO_2_ es fundamental para mantener el pH adecuado en los medios de cultivo (pH: 7,2–7,4) y permitir el correcto desarrollo embrionario [18], [19]. Actualmente, los incubadores más empleados son los llamados trigas, que emplean tres gases: CO_2_, O_2_ y N_2._ El N_2_ reduce la concentración de O_2_ a niveles de hipoxia (5%), como sucede en el útero materno, lo que se ha demostrado esencial para el desarrollo a blastocisto [20]. Además, suelen tener espacios independientes para las placas de cada paciente, sin que la apertura de uno modifique las condiciones del resto. Estos son incubadores tipo “sandwichera”, o de sobremesa, que controlan y monitorizan de forma óptima la concentración de gases y temperatura.

### Criopreservación embrionaria: vitrificación

Otro avance fundamental del laboratorio para la implantación de programas de eSET ha sido el desarrollo de la técnica de vitrificación/desvitrificación. El procedimiento consiste en la congelación/descongelación ultrarrápida mediante el intercambio de agua y sustancias crioprotectoras, evitando la formación de cristales de hielo y alcanzando tasas de supervivencia del 78–100% [21]. Esta técnica ha supuesto una auténtica revolución ya que hasta su aparición la única técnica existente era la congelación lenta, que induce la formación de cristales de hielo en el interior celular. Esto provoca, en muchos casos, que el embrión degenere siendo la tasa de supervivencia muy inferior, aproximadamente del 60%. Por otro lado, el proceso requiere mucho tiempo y aparataje más complejo que la vitrificación [22]. Así, la posibilidad de vitrificar embriones con tan buen pronóstico de supervivencia facilita la decisión de transferir uno solo por intento. Además, la vitrificación ha convertido en habitual la transferencia embrionaria en diferido en lugar de en fresco, con el objetivo de conseguir una preparación endometrial y un ambiente uterino óptimos y aumentar así el éxito del tratamiento [23].

Los avances tecnológicos expuestos han permitido que los embriones se desarrollen y criopreserven *in vitro* en condiciones idóneas y facilitan la elección de aquellos con buen potencial implantatorio, aumentando las posibilidades de éxito del eSET a la vez que se reduce la de embarazo múltiple. Tras la introducción de estos cambios en el laboratorio, la forma más recomendada de calcular porcentajes de éxito ya no es la tasa de embarazo por transferencia sino la “tasa de embarazo acumulada”, que tiene en cuenta todas las transferencias realizadas con embriones procedentes de un mismo ciclo de obtención de ovocitos, tanto en fresco como en diferido con embriones desvitrificados [23].

## Factores influyentes en la elección de eSET

### Contraindicaciones médicas para transferencia embrionaria múltiple

Aunque el riesgo materno aumenta en un embarazo múltiple en mujeres sanas, el término mSET (*medical* SET) se reserva para aquellas mujeres en las que una gestación múltiple está absoluta o relativamente contraindicada por razones médicas conocidas que interfieren con un buen resultado obstétrico, en lugar de por razones fetales [6]. Son, en consecuencia, las únicas situaciones que *obligan* a una eSET. Se debe asesorar sobre estos riesgos [6], [24]:

Contraindicaciones absolutas:

– Anomalía uterina Mülleriana congénita que suponga riesgo significativo de parto prematuro.
– Rotura o cirugía uterina previa.
– Insuficiencia ístmica.
– Síndrome de Turner.
– Enfermedad sistémica severa.
– Enfermedad psiquiátrica severa.
– Pérdida temprana previa de un embarazo múltiple.
– Diabetes insulinodependiente.

Contraindicaciones relativas:

– Antecedentes de parto prematuro en embarazo único/gemelar.
– Mujeres sin pareja.
– Parejas de mujeres.
– Mujeres de edad avanzada.
– Mujeres parapléjicas.
– Enfermedad sistémica moderada/leve.
– Enfermedad psiquiátrica moderada/leve.
– Trastornos tromboembólicos.

### Edad de la paciente

Según las recomendaciones de la ASRM, la eSET debe ser estrategia de elección en mujeres con mejor pronóstico de embarazo. Así, se recomienda a mujeres: menores de 35 años, en primer o segundo ciclo, con embarazo en ciclo previo o receptoras de óvulos, y debe considerarse en mujeres entre 35–40 años cuando se dispone de blastocistos de buena calidad [25]. En mayores de 40 años, la decisión de realizar transferencia electiva de uno o dos blastocistos depende de si se dispone de más de dos blastocistos transferibles/vitrificables, ya que aunque la tasa de nacido vivo se duplica al realizar transferencia electiva de dos blastocistos, la tasa de parto múltiple pasa del 0 al 22% [26]. En la franja de 40–43 años, la edad no es un factor predictivo de la tasa de nacidos, siendo similar la tasa acumulada en ambos casos pero con un gran aumento en la tasa de parto múltiple (0 vs. 14,9%) [27]. Ahora bien, las recomendaciones previas sobre el número de embriones a transferir deben adaptarse también a la edad ovárica de la paciente, la cual puede estar mejorada o reducida [24].

Por otra parte, en algunos países existen regulaciones específicas acerca del número de embriones a transferir en función de la edad de la paciente. Así, en Bélgica se practica el *legally enforced* SET, donde se regula por ley el número de embriones a transferir en función de la edad de la mujer y del número de ciclos realizados. Se prohíbe la transferencia embrionaria múltiple en fresco en el primer ciclo a aquellas mujeres ≤ 36 años y lo mismo se aplica para el segundo ciclo excepto que el embrión sea de baja calidad. Su cumplimiento garantiza la financiación de cada ciclo pero es obligatorio para toda paciente tratada en suelo belga, incluso si se paga los tratamientos [10].

### Estadio embrionario: Día 3 (D3) vs. Día 5 (D5)

La justificación de prolongar el cultivo embrionario y realizar la transferencia en fresco de blastocisto frente al D3 radica en mejorar la sincronía uterina/embrionaria y permitir la autoselección de embriones viables, lo que resulta en mejores tasas de embarazo clínico y nacidos vivos [17], [28], [29]. Ahora bien, no existen diferencias significativas entre las tasas de embarazo *acumulado* por ciclo realizado con blastocistos y con embriones de D3, ni en la tasa de embarazos múltiples o de abortos [17]. Además, los embriones de D3 con alta calidad (7–8 células, mínima fragmentación y sin multinucleación) tienen altas tasas de implantación [30]. Así, la tasa de embarazo clínico tras transferir dos embriones de buena calidad en D3 es similar a la alcanzada tras una transferencia electiva de blastocisto, aunque a expensas de una alta tasa de embarazo gemelar (43,26 vs. 0,6%) [31]. Por tanto, no debe decidirse qué día realizar la transferencia hasta conocer el número y la calidad de los embriones en D3.

La eSET debe considerarse también en la transferencia de embriones criopreservados en D3, especialmente tras cultivo de los embriones desvitrificados hasta blastocisto, ya que la tasa de embarazo múltiple, aunque inferior que tras transferencia en fresco, es significativa.

Por todo ello, se concluye que la eSET es valorable en ambos estadios embrionarios debiendo considerarse el factor de esterilidad y la calidad embrionaria, además del rendimiento del programa de criopreservación de la clínica en el caso de embriones criopreservados [25]. Por otro lado, se debe informar a las pacientes que deseen una transferencia en D5 de que tienen mayor riesgo de no disponer de embriones para transferir y de tener menor número disponible para criopreservar, ya que no todos los embriones de D3 alcanzan el estadio de blastocisto. Asimismo, deben conocer que existen datos en modelos animales de que las condiciones del cultivo largo inducen alteraciones epigenéticas por modulación de la metilación del ADN, resultando en un incremento de las mismas y en defectos de la impronta genética [23].

### Número de embriones disponibles de buena calidad

Según las recomendaciones de la ASRM, la eSET debe realizarse solamente en aquellas mujeres con más de un embrión disponible para transferir de buena calidad y en aquellas con embriones sobrantes para criopreservar [25]. Cuando solo hay un blastocisto de buena calidad disponible, transferir dos blastocistos utilizando un segundo embrión de pobre calidad frente a transferir solamente el de buena calidad no aumenta la tasa de nacidos vivos pero aumenta significativamente el riesgo de nacidos múltiples, tanto en transferencias en fresco como diferidas [32]. Estos resultados confirman estudios previos y apoyan el utilizar eSET cuando hay al menos un embrión de alta calidad disponible [23], [33].

Valorando los dos apartados anteriores, se ha demostrado en estudios controlados y aleatorizados que la transferencia de un único blastocisto en fresco, de calidad morfológica excelente, genera una tasa de embarazo evolutivo por ciclo significativamente superior a la obtenida tras la transferencia de embriones de D3 [34], [35].

### Valoración embrionaria morfológica convencional vs. “Time-lapse”

El método más extendido para identificar los embriones que potencialmente darán una gestación se basa en una valoración morfológica y categorización según parámetros clave del desarrollo como: número y simetría celular, multinucleación, fragmentación y velocidad de desarrollo, etc. En España, la Asociación para el Estudio de la Biología de la Reproducción (ASEBIR) ha establecido un sistema de clasificación morfológica basado en evidencia científica, opinión de expertos, controles de calidad externos, encuestas y estudios multicéntricos; que relaciona determinadas características morfológicas con una probabilidad estimada de implantación [36]. Por otra parte, la valoración de la morfología embrionaria hasta hace poco únicamente podía realizarse mediante observación directa en microscopio invertido. Es un método sencillo pero solo aporta información del momento en que se realiza la observación y es susceptible de variación inter-observador y, por tanto, subjetivo [37]. La irrupción de la tecnología time-lapse en el laboratorio de TRA ha cambiado esta práctica. Consiste en un incubador con microscopio y cámara integrados, que permiten la monitorización continua y no invasiva de los embriones. Así se evita extraerlos para observarlos en el microscopio, lo que modifica las condiciones de cultivo y puede alterar su desarrollo. Esta tecnología ha permitido desarrollar un método de valoración embrionaria más objetivo y fiable ya que, debido a la gran cantidad de imágenes generadas, aporta información completa sobre el desarrollo morfológico del embrión [38]. Además, ofrece la ventaja de poder seleccionar el embrión tanto por criterios morfológicos como dinámicos o morfocinéticos [19]. Por tanto, el time-lapse ofrece una potencial ventaja para realizar eSET gracias a la valiosa información obtenida mediante la monitorización ininterrumpida del desarrollo embrionario [39].

Numerosos estudios han demostrado que el cultivo de embriones en incubadores time-lapse no afecta a su desarrollo en comparación con incubadores convencionales [40]. Por el contrario, proporciona condiciones seguras y estables que favorecen su desarrollo. Por otro lado, a pesar de que su principal desventaja es el mayor coste del cultivo, es una importante fuente de información que puede ayudar al embriólogo en la difícil tarea de seleccionar el embrión de mejor calidad y con mayor capacidad de implantación, gracias a programas y algoritmos predictivos basados en más de 70 parámetros morfocinéticos. Solo con tecnología time-lapse es posible la observación de eventos específicos del desarrollo como, por ejemplo, la multinucleación, la división directa (divisiones de una a tres células) y la división inversa (fusión celular). La observación de estos eventos es de suma utilidad ya que se asocian con un bajo potencial implantatorio [41]. Distintos trabajos han comparado los resultados obtenidos mediante una estrategia de selección embrionaria basada en la morfocinética y una basada en observaciones morfológicas puntuales. Según algunos autores, la estrategia morfocinética mejora la tasa de implantación, de embarazo y, en definitiva, los resultados reproductivos [42]. Sin embargo, según otros no hay relación significativa entre la mejoría de estos resultados y la utilización de incubadores time-lapse [43]. Así pues, no existe suficiente evidencia de que las tasas de éxito sean significativamente diferentes entre el cultivo y valoración embrionarios mediante métodos convencionales y mediante time-lapse que permita utilizar esta tecnología para decidir la práctica de una eSET [44].

### Test genético preimplantacional de aneuploidías (PGT-A)

La tasa de aneuploidía ovocitaria aumenta con la edad materna y en consecuencia también ocurre lo mismo en los embriones, afectando a las tasas de embarazo y aborto [45]. Gracias al PGT-A es posible seleccionar aquellos embriones con una dotación cromosómica correcta y descartar aquellos aneuploides, aumentando así la tasa de nacido vivo sano incluso transfiriendo un único embrión euploide [46]. Para realizar el PGT-A debe emplearse una tecnología segura que no dañe al embrión. Hoy en día la técnica más extendida es la biopsia de trofoectodermo en estadio de blastocisto. De esta forma se obtiene un mayor número de células para el estudio genético, lo que mejora la sensibilidad de la técnica al reducir considerablemente el número de embriones sin diagnóstico (<5%) y se evita manipular la masa celular interna, lo que reduce el riesgo del procedimiento. Además, permite seleccionar embriones euploides, y por tanto con mejor potencial implantatorio, incluso tras biopsia y vitrificación del blastocisto en una o dos ocasiones [47]. En caso de tener que revitrificar tras una segunda biopsia embrionaria sí se reduce la tasa de embarazo [47]. Por ello, hoy en día, las guías que recomiendan un número de embriones a transferir ya no solo se basan en edad de la mujer, número de embriones disponibles o estadio embrionario, sino también en la ploidía del embrión (9,46). Muchos estudios defienden el uso del PGT-A para aumentar la utilización de eSET en pacientes sometidas a FIV [46], ya que la combinación de ambas estrategias aumenta la tasa de recién nacido y disminuye la incidencia de gestaciones múltiples [48]. Así, su aplicación en mujeres con edades comprendidas entre 35–40 años parece mejorar las tasas de embarazo clínico y nacido vivo, ayudando por tanto a mitigar los efectos negativos de la edad materna en los resultados, aunque este incremento desaparece cuando se calculan tasas acumuladas de éxito [49], [50]. Además, se ha observado que las tasas de implantación, embarazo clínico y nacido vivo se mantienen estables independientemente de la edad con el uso de eSET más PGT-A y no presentan diferencias significativas con las obtenidas en ciclos de transferencia embrionaria doble [51]. Finalmente, es importante considerar que el cultivo hasta blastocisto en incubadores time-lapse combinado con selección embrionaria mediante PGT-A mejora significativamente la tasa de embarazo clínico frente a incubadores convencionales [52]. Sin embargo, aunque el PGT-A puede beneficiar a ciertos grupos de pacientes no existen evidencias suficientes para su aplicación universal, ya que aumenta notablemente el coste del tratamiento especialmente en grupos de mujeres jóvenes, sin olvidar su aspecto invasivo.

## Tasas de éxito de SET

### Modelo comparativo: 2xeSET vs. 1xDET (*Double Embryo Transfer*)

Las tasas de éxito tras transferir un solo embrión, de forma electiva o no, son por lo general inferiores (10–40%) a las esperables tras transferir dos o más embriones [53], [54]. No obstante, algunos autores defienden que al realizar una eSET estas tasas pueden equiparse a las obtenidas tras una transferencia embrionaria múltiple no electiva [55]. Y que es posible conseguir resultados similares incluso en pacientes de peor pronóstico como mujeres de edad avanzada [27]. Ciertos autores, por el contrario, cuestionan la eficacia de la SET en este grupo de pacientes [56]. Por otra parte, se ha demostrado de forma consistente que, en caso de no conseguir embarazo tras una SET, las tasas de gestación evolutiva y nacido vivo se equiparan tras la subsecuente transferencia de un embrión desvitrificado a las obtenidas tras una DET [57]. Asimismo, se ha evidenciado que la SET permite el descenso de las elevadas tasas de gestación múltiple tras transferencia embrionaria múltiple (20–50%) hasta equipararse a las tasas de gestación múltiple espontáneas (3%) [57], [58]. Se concluye que la eficacia de dos SET es equivalente, o incluso superior en el caso de eSET, a la de una DET, pudiendo evitar además los riesgos asociados con el embarazo múltiple.

## Coste-eficacia de eSET

### Costes en salud del embarazo múltiple para la mujer y la descendencia

La gestación múltiple es considerada la principal complicación iatrogénica de las TRA debido a su asociación con diversos sucesos adversos, que pueden afectar tanto a la madre como a la descendencia [13]. Las principales consecuencias negativas están relacionadas con la prematuridad de los recién nacidos: restricción del crecimiento, ictericia o complicaciones respiratorias; siendo la probabilidad de parto prematuro en una gestación múltiple 5–9 veces superior que en una gestación única [59]. Se ha descrito también un aumento en la incidencia de complicaciones maternas: preeclampsia, diabetes gestacional, placenta previa, desprendimiento de placenta, rotura prematura de membranas y cesárea [59], [60], [61]. Además, este aumento ocurre también en mujeres de edad avanzada, pudiendo tener consecuencias aún más severas que en pacientes jóvenes [62]. Así pues, la gestación múltiple es una reconocida causa de aumento de morbimortalidad materno-fetal.

### Costes económicos y sociosanitarios del embarazo múltiple

La estrategia de SET requiere, según los estudios publicados, mayor número de transferencias embrionarias para conseguir embarazo que la de transferencia embrionaria múltiple [57]. Esto, a su vez, implica mayor gasto asociado a las TRA: más ciclos de estimulación hormonal, transferencia embrionaria y vitrificación/desvitrificación. Por otro lado, las gestaciones y partos múltiples acarrean importantes costes económicos a nivel sanitario, hasta 2–7 veces superiores a los de carácter único [63]. Si se incluye en este cálculo la atención pediátrica del primer año de vida, los costes económicos de un parto múltiple suponen hasta 20 veces los de uno único [64].

Según el país, los costes sanitarios derivados de las TRA son asumidos por el sistema nacional de salud o por los pacientes, lo que inevitablemente influye en la elección de estrategia de transferencia embrionaria. En las gestaciones múltiples existen además otros gastos adicionales a largo plazo que deben considerarse, como son: ropa, transporte, comida, escolarización, etc. [54]. Tras analizar los costes y resultados derivados de una estrategia de DET frente a una de eSET, Fiddelers y colaboradores [65] concluyeron que la DET es la estrategia más costosa desde un punto de vista económico y que la eSET es la estrategia más coste-efectiva, siempre que se incluya más de un ciclo de transferencia embrionaria [65]. Ahora bien, la tasa de embarazo múltiple tras transferir dos o más embriones disminuye según aumenta la edad de la mujer con lo que la transferencia embrionaria múltiple podría ser considerada coste-efectiva en algunas pacientes de edad avanzada [66].

Las gestaciones múltiples tienen, por tanto, un elevado coste sociosanitario que podría paliarse mediante la aplicación de la SET sin disminuir la eficacia de las TRA, siempre que se considere el éxito como tasa acumulada [67].

### Costes psicosociales del embarazo múltiple para los pacientes

Existen determinados factores psicosociales de riesgo asociados a la gestación y el parto que deben considerarse. Según diversos estudios poblacionales, las mujeres tienen más probabilidades de padecer síntomas de depresión y episodios de estrés post-parto en caso de parto múltiple que de parto único. No existen, sin embargo, diferencias significativas en la probabilidad de padecer estos síntomas entre las mujeres que han conseguido el embarazo espontáneamente y las que lo han conseguido mediante TRA [68].

Asimismo, algunos autores señalan la existencia de más problemas conyugales y una pérdida de productividad, materializada en un índice superior de bajas laborales y una menor probabilidad de conseguir empleo remunerado, en el caso de las madres que afrontan un parto múltiple en lugar de uno único [54], [63]. Estos datos hacen aconsejable reforzar las estrategias de atención psicológica y cuidado a la embarazada en caso de gestación múltiple.

## Puesta en práctica de eSET

### Educación y asesoramiento

Pese a los riesgos asociados a las gestaciones y partos múltiples, hasta un 80% de pacientes desean como primera opción una transferencia embrionaria múltiple [54]. Las razones principales son: desconocimiento de dichos riesgos, deseo de máxima probabilidad de éxito, dificultades económicas para costear las TRA o bien la idea de satisfacer en un único intento el anhelo de una ma/paternidad numerosa. La información a los pacientes haciendo énfasis en: concepto de tasa de éxito acumulada, riesgos para la salud, así como implicaciones económicas y emocionales de una gestación múltiple, ha demostrado efectividad en el aumento de elección de SET [14]. El uso de herramientas predictivas personalizadas sobre el ciclo de FIV y la probabilidad de embarazo múltiple han demostrado también ser útiles, al percibirse como fuente objetiva de información [54].

### Políticas económicas y sociales de incentivación

Los gobiernos de determinados países como Turquía, Suecia, Dinamarca, Bélgica, Nueva Zelanda o Canadá, han optado por establecer o incentivar una política nacional de SET. El objetivo de estas políticas es doble: evitar riesgos para la salud de madres y recién nacidos y disminuir los elevados costes sanitarios asociados a las gestaciones múltiples. Las medidas abarcan desde la prohibición de transferencias embrionarias múltiples para la mayoría de pacientes hasta la limitación en el número de embriones transferidos para poder beneficiarse de la financiación de todo o parte del tratamiento. Dichos países han logrado aumentar de este modo las cifras de SET y disminuir las de gestación múltiple, hasta casi un 80% y un 5% respectivamente en el caso de Australia, manteniendo estables las tasas de gestación clínica y recién nacido vivo de forma acumulada [54].

Se demuestra así que, junto a una intervención educacional, el establecimiento de políticas de SET en determinados escenarios clínicos de buen pronóstico y la disposición de ayudas económicas son capaces de incentivar la elección de SET en países culturalmente tan distintos como EE.UU., Japón o Nueva Zelanda [14]. Los estímulos económicos pueden consistir en mayor disponibilidad de fondos públicos, cobertura total/parcial de los tratamientos por compañías aseguradoras o campañas promocionales de los propios centros de RHA como gratuidad de la criopreservación y/o criotransferencia embrionarias. No obstante, es interesante destacar que estas medidas no parecen tener el mismo efecto estimulante en aquellos países en los que existe la posibilidad de cubrir parcial o completamente las TRA con fondos públicos, como es el caso de Dinamarca o Suecia [14].

## Conclusiones

Más de siete millones de niños han nacido en todo el mundo como resultado de las TRA. Desde los inicios, el elevado porcentaje provocado de embarazos múltiples en RHA ha supuesto un importante dilema, debido al mayor riesgo de parto prematuro y la mayor morbimortalidad asociadas, tanto para la madre como para la descendencia.

Durante los últimos años se han desarrollado importantes avances en el campo de la medicina reproductiva, cuya aplicación ha derivado en una notable mejoría de las tasas de éxito. Destacan, entre ellos, las innovaciones en el laboratorio de RHA como: cultivo hasta blastocisto, criopreservación mediante vitrificación, tecnología “time-lapse” o PGT-A. Estas técnicas favorecen la aplicación de la SET, incluso en pacientes de peor pronóstico, sin tener que renunciar a unas tasas de éxito óptimas.

Sin embargo, y a pesar de los continuos avances técnicos, seguirán existiendo diferencias, potencialmente significativas, entre las tasas de éxito por SET y por transferencia embrionaria múltiple mientras se sigan expresando como tasas por transferencia. En la actualidad, es necesario referirse a tasas acumuladas, en las cuales se incluyen todas las transferencias realizadas con embriones procedentes de un mismo ciclo, tanto en fresco como en diferido.

La presente revisión bibliográfica pretende ayudar a los profesionales a tomar conciencia del relevante problema de salud pública que supone, hoy en día, el embarazo múltiple en RHA y a fomentar el uso de la SET como estrategia para conseguir un recién nacido vivo sano y a término en casa.
